# Pan-cancer analysis of Arp2/3 complex subunits: focusing on ARPC1A’s role and validating the ARPC1A/c-Myc axis in non-small cell lung cancer

**DOI:** 10.3389/fimmu.2024.1491910

**Published:** 2025-01-10

**Authors:** Chenkang Zhou, Yuxin Chen, Shuhui Chen, Lijuan Hu, Junjun Wang, Yumin Wang

**Affiliations:** ^1^ Cixi Biomedical Research Institute, Wenzhou Medical University, Wenzhou, Zhejiang, China; ^2^ Department of Laboratory Medicine, The First Affiliated Hospital of Wenzhou Medical University, Wenzhou, Zhejiang, China

**Keywords:** ARPC1A, Arp2/3 complex subunits, pan-cancer analysis, biomarker, immune infiltration

## Abstract

**Background:**

The Arp2/3 complex is a key regulator of tumor metastasis, and targeting its subunits offers potential for anti-metastatic therapy. However, the expression profiles, prognostic relevance, and diagnostic value of its subunits across cancers remain poorly understood. This study aims to investigate the clinical relevance of Arp2/3 complex subunits, particularly ARPC1A, in pan-cancer, and to further analyze the potential biological mechanisms of ARPC1A, as well as its association with immune infiltration and chemotherapy drug sensitivity.

**Methods:**

To explore the differential expression of Arp2/3 complex subunits and their clinical relevance across cancers, we analyzed data from TCGA and GTEx databases. The relationship between ARPC1A and immune infiltration, as well as its interactions with functional proteins, was examined using the TCPA and TIMER2.0 databases. Gene Set Enrichment Analysis (GSEA) was performed to identify ARPC1A-associated signaling pathways. Chemotherapy drug sensitivity correlated with ARPC1A expression was assessed using CellMiner, GDSC, and CTRP databases. The effect of ARPC1A on c-Myc expression was validated by quantitative PCR (qPCR) and Western blot. Finally, the biological role of ARPC1A in non-small cell lung cancer (NSCLC) cells was further validated using CCK-8, EdU incorporation, colony formation, and Transwell assays.

**Results:**

The Arp2/3 complex subunits, particularly ARPC1A, are frequently overexpressed in a majority of cancers, correlating with poor prognostic outcomes and demonstrating significant diagnostic utility. Copy number variations may play a role in the dysregulation of Arp2/3 complex subunit expression. The small molecule X4.5.dianilinophthalimide has shown promise as a targeted therapeutic agent in a pan-cancer context. Functional predictions indicate that ARPC1A is implicated in oxidative phosphorylation pathways and cell proliferation-related signaling pathways, including those mediated by MYC, with ASNS potentially acting as an upstream regulator. Furthermore, ARPC1A has been implicated in the resistance to chemotherapy drugs, including gefitinib. *In vitro* experiments corroborate that ARPC1A may enhance malignant phenotypes in non-small cell lung cancer (NSCLC) cells through the regulation of c-Myc expression.

**Conclusion:**

Our study offers novel insights into targeting Arp2/3 complex subunits as an anti-cancer strategy and underscores the potential of ARPC1A as a novel biomarker for tumor diagnosis, prognosis, and the prediction of immune therapy responses.

## 1 Introduction

The Arp2/3 complex, essential for actin polymerization and nucleation, comprises seven distinct subunits in human cells: the actin-related proteins ARP2 and ARP3, and five ARPC subunits (ARPC1-5). Among these, Arp3, ARPC1, and ARPC5 subunits each have distinct isoforms. For instance, ARPC1 comprises two isoforms: ARPC1A and ARPC1B (1). Extensive research reveals that ARP2/3 complex overexpression in multiple cancers is linked to poor outcomes and promotes tumor metastasis through actin cytoskeleton regulation. It regulates tumor cell migration, invasion, and metastasis by influencing the actin cytoskeleton (2).

Notably, targeting Arp2/3 subunits may provide new strategies for anti-metastatic therapy (3, 4) and overcoming radiotherapy resistance (5). ​​Phenylpiperazine specifically inhibits cancer cell invasion and migration by targeting ARPC2, with no impact on normal cells (3). Additionally, in GBM, ARPC1B knockdown suppresses the MES phenotype and enhances GSC radiosensitivity (5). The study by Huang et al. preliminarily elucidated the expression levels of Arp2/3 complex subunits across various cancers (6); however, the databases used were somewhat limited, and the clinical relevance and mechanisms of expression dysregulation of all subunits were not further analyzed. Therefore, in this study, we aimed to explore novel anticancer strategies targeting Arp2/3 complex subunits. To this end, we comprehensively utilized multiple databases from a pan-cancer perspective to investigate the expression profiles, potential dysregulation mechanisms, and identified small-molecule compounds targeting the respective subunits. Additionally, we further analyzed the potential value of each subunit as a diagnostic and prognostic biomarker across various cancers.

Chen et al. demonstrated ARPC1A as a potential biomarker for prostate cancer. They showed that ARPC1A facilitates cytoskeletal formation, invasion, and migration of prostate cancer cells. Additionally, glutamine metabolism was found to upregulate its expression (7). However, ARPC1A’s role across different cancers requires further clarification. Our study leveraged public databases and bioinformatics tools to comprehensively explore the biological mechanisms of ARPC1A in a pan-cancer context, its association with the immune microenvironment and chemotherapy sensitivity, and its potential as a biomarker for predicting immunotherapy response. We also validated the regulatory effects of ARPC1A on the malignant phenotype of NSCLC and c-Myc expression *in vitro*. The findings underscore ARPC1A’s significance in multiple cancers, pointing to the need for in-depth studies on its molecular mechanisms, targeted treatments, and combination therapy approaches.

## 2 Results

### 2.1 Expression profiles across cancers and diagnostic/prognostic significance of Arp2/3 complex subunits

**Figure 1:**
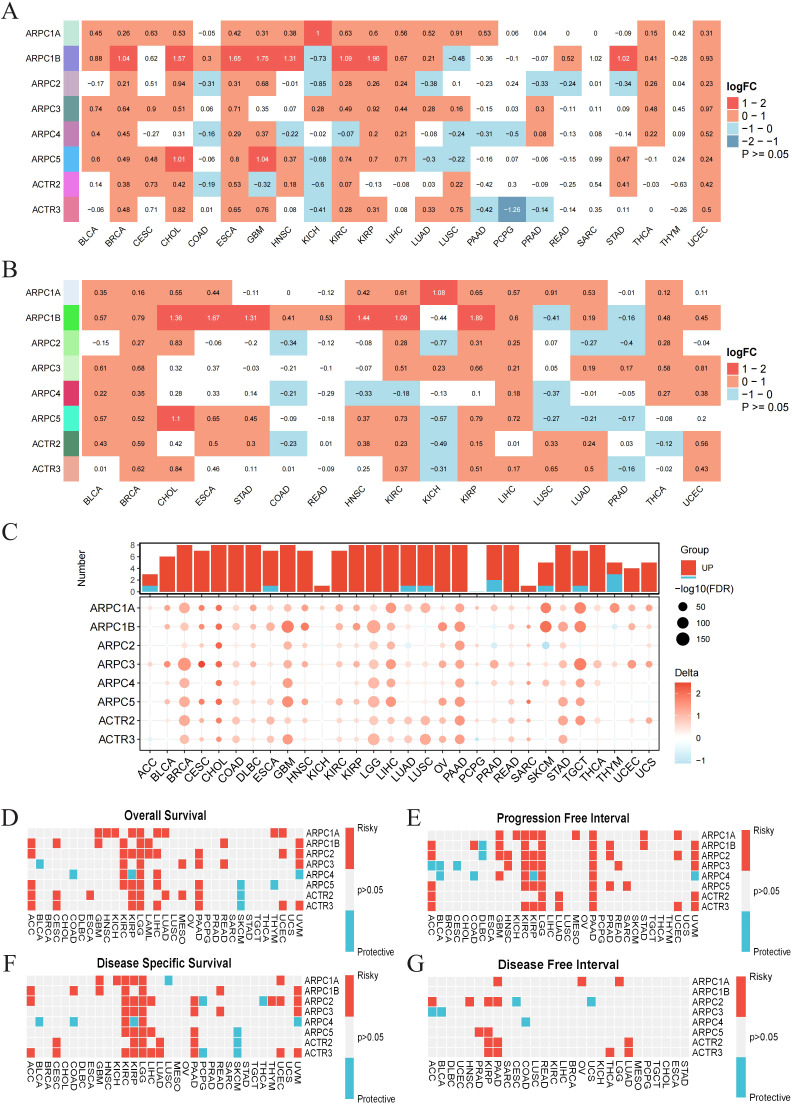
Expression profiles across cancers and diagnostic/prognostic significance of Arp2/3 complex components. **(A)** Differential expression of Arp2/3 complex subunits in tumor versus normal tissues based on TCGA data. Red indicates positive differential expression with a Wilcoxon rank-sum test p-value < 0.05, blue indicates negative differential expression with a p-value < 0.05, and white indicates a p-value > 0.05. The color intensity reflects the magnitude of absolute differential expression. **(B)** Expression differences of Arp2/3 complex subunits between paired tumor and normal tissues from the TCGA database. **(C)** Differences in Arp2/3 complex subunit expression between tumor and normal tissues, based on the TCGA and GTEx databases. Bubble size indicates the significance of the difference, with larger bubbles representing greater significance. **(D–G)** Heatmap showing the correlation between Arp2/3 complex subunits’ expression levels and four survival outcomes: overall survival (OS), disease-specific survival (DSS), disease-free interval (DFI), and progression-free interval (PFI). Red indicates a hazard ratio > 1, reflecting increased risk, while blue indicates a hazard ratio < 1, reflecting decreased risk.

To elucidate the expression patterns of Arp2/3 complex subunits in pan-cancer, we first performed differential analysis using the TCGA database (**Figure 1A**) and paired differential analysis (**Figure 1B**). The results are shown as a heatmap. Both analyses showed that Arp2/3 complex components’ mRNA levels are notably increased in BRCA, with ARPC1A and ARPC1B upregulated across most cancer types. Additionally, the GTEx database was used with TCGA to increase the number of normal samples, further validating these results (**Figure 1C**).

We then assessed the diagnostic and prognostic value of Arp2/3 complex subunits across various cancers. ROC curves based on TCGA samples showed that most Arp2/3 complex subunits have high diagnostic value (AUC > 0.7) for various cancers. Specifically, ARPC1A mRNA levels had high sensitivity and specificity for diagnosing 12 cancer types (AUC > 0.7). With increased normal sample sizes from the GTEx database, ARPC1A showed robust diagnostic value across 20 cancer types, including LUAD (**Supplementary Figure 1**).

To assess prognostic significance, we examined the relationship of Arp2/3 complex components’ mRNA levels with survival metrics—such as overall survival (OS), disease-specific survival (DSS), disease-free survival (DFS), and progression-free interval (PFI)—across 32 cancer types. Pan-cancer survival analysis indicates the potential of Arp2/3 complex components as prognostic biomarkers. ARPC1A, in particular, is a risk factor for multiple cancers, notably LGG, where it is identified as a risk factor across all survival periods predicted by univariate Cox analysis. Furthermore, all Arp2/3 complex subunits are risk factors for LGG prognosis in OS, PFI, and DSS (**Figures 1D–G**). In conclusion, these findings emphasize the connection of Arp2/3 complex components, particularly ARPC1A, with cancer progression, reinforcing their potential as biomarkers for cancer diagnosis and prognosis.

### 2.2 Genetic alteration analysis of Arp2/3 complex subunits

**Figure 2:**
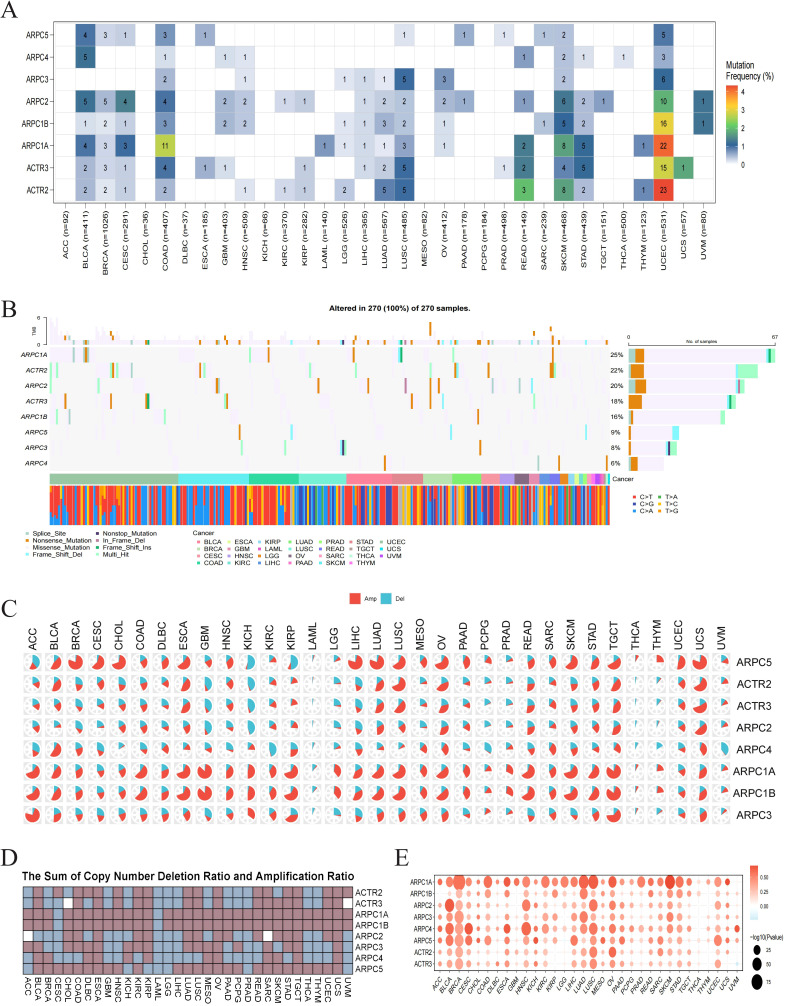
Genetic alteration analysis of Arp2/3 complex subunits. **(A)** Single nucleotide variation (SNV) frequency in Arp2/3 complex subunits. The color scale on the right represents the proportion of mutant patients relative to the total number of patients. Numbers within the colored blocks indicate the count of mutated samples; ‘0’ denotes no mutations in the coding region of that gene, and blocks without numbers indicate no mutations in any gene region. Color intensity ranges from red (higher number of mutated samples) to white (lower number of mutated samples). **(B)** Mutational distribution of Arp2/3 complex subunits and associated SNV types across cancers. **(C, D)** Copy number amplification and deletion rates of Arp2/3 complex subunits across cancers. In pie chart **(C)**, the red segment represents the proportion of amplifications, while the blue segment denotes the proportion of deletions. Heatmap **(D)** shows the combined amplification and deletion ratios. **(E)** Correlation between CNV and Arp2/3 complex subunit mRNA expression. The color of each bubble indicates the direction of the correlation between specific gene mRNA expression and copy number variation, with red signifying positive correlation and blue signifying negative correlation. Bubble size reflects the significance of the correlation (p value), with larger bubbles indicating greater significance and smaller p values.

To understand the causes of Arp2/3 component dysregulation across cancers, we examined their gene mutations and copy number changes. SNP data related to Arp2/3 complex subunits were utilized to assess the frequency and types of genetic alterations across different cancer subtypes. Notably, high frequencies of SNV mutations were observed in UCEC, SKCM, and COAD (**Figure 2A**). Variant analysis revealed that missense mutations are the predominant SNP type for these subunits, with ARPC1A exhibiting the highest mutation frequency (**Figure 2B**). Moreover, ARPC1A, ARPC1B, and ARPC5 demonstrated significant copy number amplification in most cancer types (**Figures 2C, D**). We then assessed how copy number alterations related to the mRNA levels of Arp2/3 complex components. The findings showed that mRNA levels positively associated with copy number values in cancers such as BLCA, BRCA, LUAD, and LUSC. Notably, ARPC1A displayed a consistent and significant positive correlation across most tumors (**Figure 2E**). These results suggest that the imbalance in the levels of Arp2/3 complex components, particularly ARPC1A, may be driven by genetic alterations across various cancer types.

### 2.3 Potential therapeutic strategies targeting Arp2/3 complex subunits

**Figure 3:**
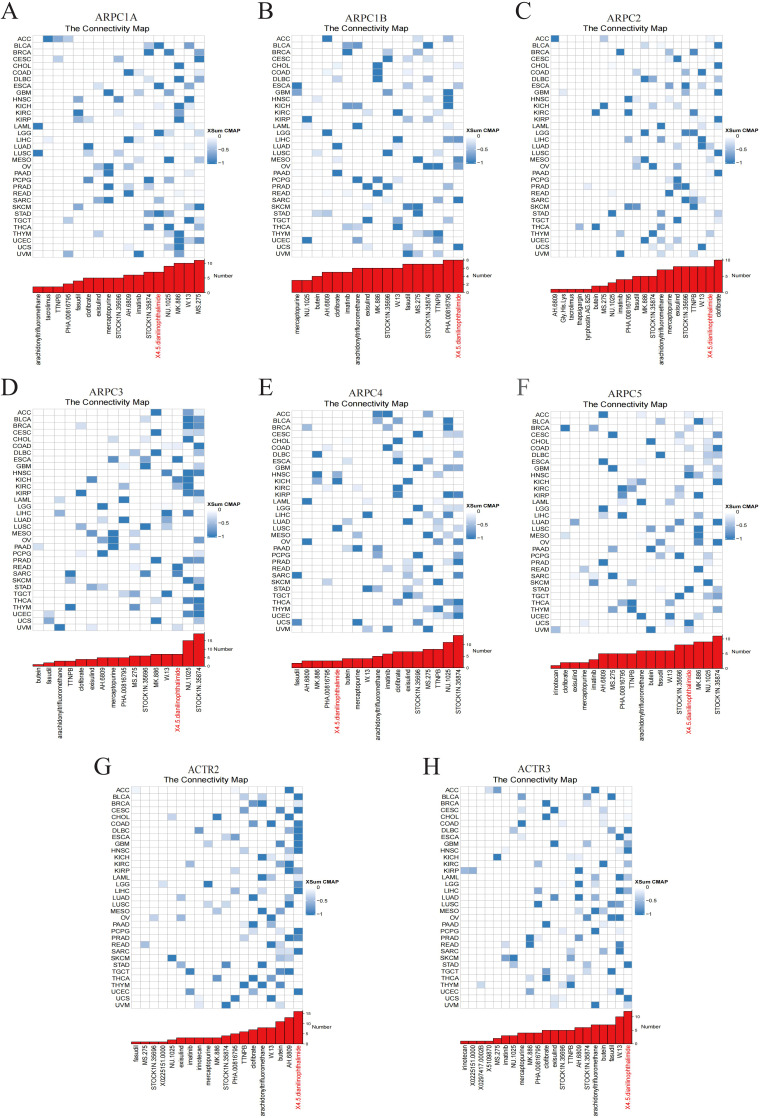
Potential small molecules targeting Arp2/3 complex subunits. **(A–H)** The blue color gradient on the right indicates the correlation between gene expression levels and small molecule compounds, with darker blue representing stronger negative correlations. Each row corresponds to a type of cancer, and each column represents a small molecule compound.

We employed CMap analysis to identify potential therapies targeting Arp2/3 complex subunit-mediated tumor promotion. Gene signatures associated with Arp2/3 subunits were constructed by comparing patients with high versus low expression of these subunits across various cancer types, including the 150 most upregulated and 150 most downregulated genes. Applying the eXtreme Sum (XSum) approach, we assessed ARPC1A-related characteristics against CMap gene profiles, resulting in correlation metrics for 1,288 substances. Interestingly, X4.5.dianilinophthalimide exhibited low correlation metrics across most malignancies, indicating its capacity to block Arp2/3 complex-driven cancerous effects (**Figures 3A–H**).

### 2.4 Exploring the potential biological role of ARPC1A in pan-cancer

**Figure 4:**
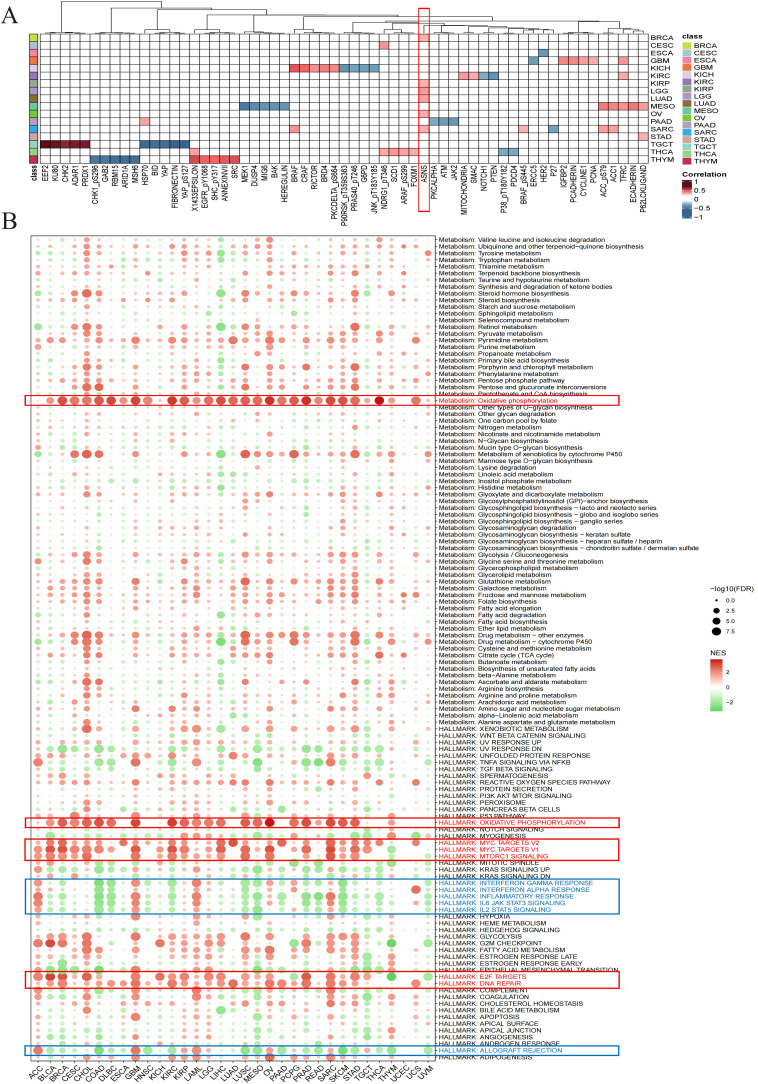
Exploring the potential biological role of ARPC1A in pan-cancer **(A)** Heatmap showing the correlation between ARPC1A and proteins in the TCPA database. The color intensity indicates the strength of the correlation, with red hues representing stronger positive correlations and blue hues indicating stronger negative correlations. **(B)** Differential enrichment analysis of ARPC1A in 50 HALLMARK gene sets and 83 metabolic gene sets.

Gene set pathway activity scoring revealed significant differences in Arp2/3 complex subunit activity between tumor and normal tissues (**Supplementary Figure 2**), indicating its potential involvement in multiple oncogenic pathways. Given ARPC1A’s high expression across cancer types and its substantial diagnostic value relative to other subunits, it was selected for further investigation. Initially, we evaluated the relationship of ARPC1A mRNA levels with a range of functional proteins in different cancers by utilizing the TCPA database. A strong positive correlation between ARPC1A mRNA levels and ASNS was observed across six cancer types, including LUAD (**Figure 4A**). Given that recent studies have shown glutamine metabolism upregulates ARPC1A in prostate cancer (7) and ASNS is a key enzyme in glutamine metabolism, it may mediate ARPC1A upregulation.

To investigate the biological roles of ARPC1A, we performed GSEA after categorizing tumors into groups with elevated and reduced ARPC1A expression to assess the stimulation or suppression of 50 hallmark gene sets and 83 metabolic gene sets. Heatmap showed that substantial activation of oxidative phosphorylation and signaling pathways related to cell proliferation, such as DNA repair, TORC1, MYC and E2F, was correlated with elevated ARPC1A expression in cancer patients (**Figure 4B**). Recent studies suggest that certain tumor subtypes, such as SWI/SNF-mutant lung cancer cells (8), KRAS-driven lung and pancreatic cancer cells (9, 10), and acute myeloid leukemia (AML) cells, rely primarily on oxidative phosphorylation instead of glycolysis for proliferation and survival (11), indicating ARPC1A may promote tumor growth through this pathway. Moreover, elevated ARPC1A expression was linked to a marked downregulation of immune-related pathways, including those involving interferon (IFN)-γ, IFN-α, IL-6, IL-12, inflammation and alloimmune rejection. The findings above suggest that ARPC1A may contribute to immune suppression in these cancers. In summary, our results highlight a strong association between elevated ARPC1A expression, oxidative phosphorylation metabolism, proliferation, and immune suppression in cancer.

### 2.5 ARPC1A is linked to immune cell infiltration across cancers

Given that prior GSEA analysis (**Figure 4B**) suggested ARPC1A may be involved in immune suppression, we compared immune scores among patients with elevated and reduced ARPC1A expression across 32 cancer types. In COAD, LUSC, PAAD, READ, STAD, THCA, and THYM, patients exhibiting high levels of ARPC1A expression showed notably reduced immune scores when compared to those with lower ARPC1A expression (**Supplementary Figure 3**). Conversely, in CESC, LGG, TGCT, and UVM, immune scores were positively correlated with ARPC1A expression (**Supplementary Figure 3**). T Such outcomes are in agreement with the GSEA analysis, suggesting that ARPC1A may lead to reduced immune infiltration in COAD, LUSC, PAAD, READ, STAD, THCA, and THYM, whereas it might increase immune infiltration in CESC, LGG, TGCT, and UVM.

**Figure 5:**
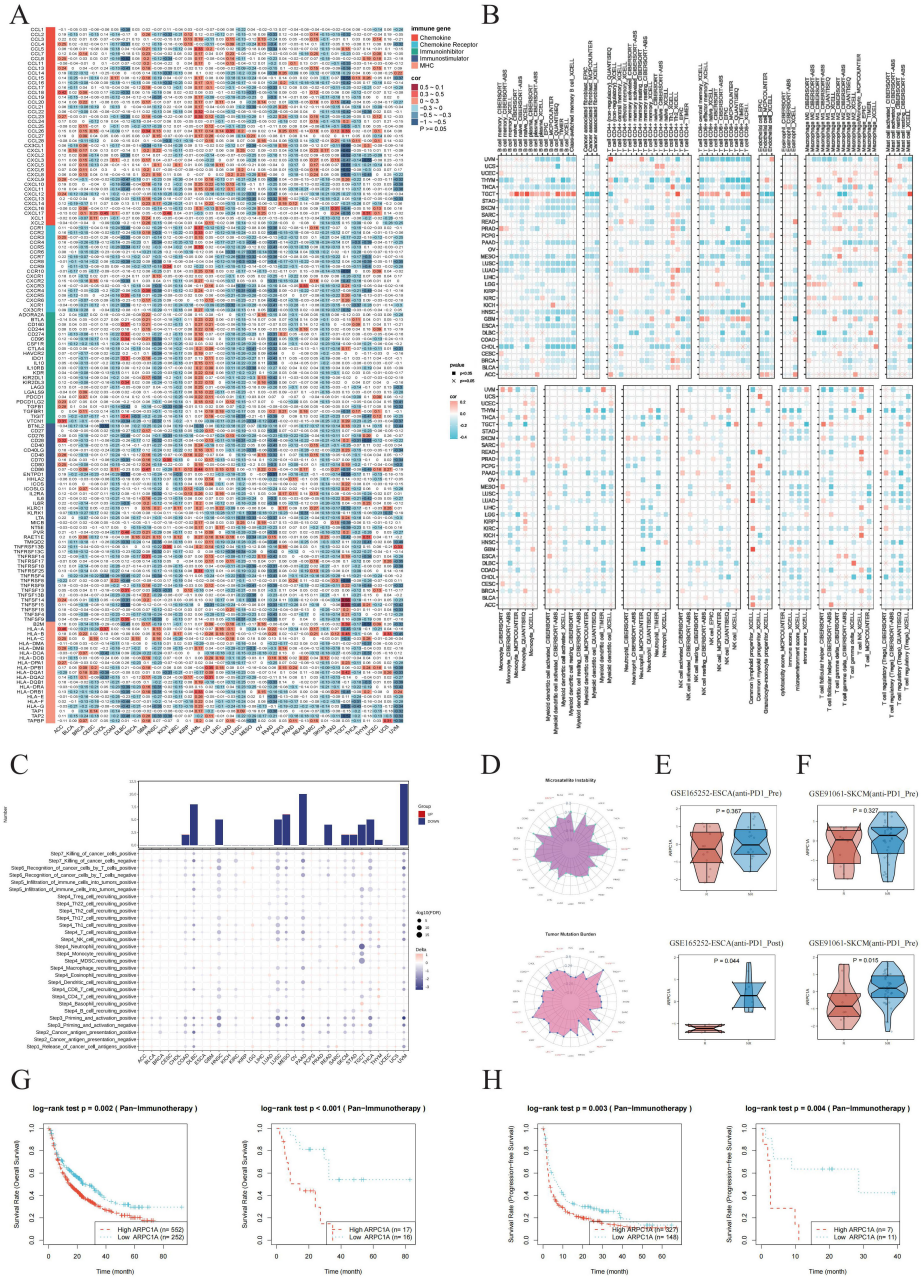
ARPC1A is linked to immune cell infiltration across cancers. **(A)** Heatmap showing the correlation between ARPC1A mRNA expression and the expression levels of chemokines, chemokine receptors, immune inhibitors, immune stimulators, and MHC genes. **(B)** Correlation assessment of ARPC1A mRNA expression with tumor immune infiltration based on seven algorithms. **(C)** Heatmap illustrating the TIP scores between high and low ARPC1A expression groups. TIP scores reflect the level of immune cell infiltration and the overall immune activity within the tumor microenvironment. The results show that high ARPC1A expression is associated with lower TIP scores, indicating reduced immune cell infiltration and a weaker immune response. This suggests that high ARPC1A expression may promote immune evasion, potentially reducing the effectiveness of immunotherapy. **(D)** Radar plot displaying the correlation of ARPC1A mRNA expression with microsatellite instability (MSI, left) and tumor mutational burden (TMB, right) across various cancer types. **(E, F)** Expression differences of ARPC1A between the immune response responders and non-responders before and after immunotherapy in ESCA and SKCM. The x-axis represents the response groups, with “R” for responders and “NR” for non-responders. The y-axis represents the z-score values of ARPC1A expression. “Pre” refers to before immune response, and “Post” refers to after immune response. **(G, H)** The relationship between ARPC1A expression and OS and PFS in different SKCM immune therapy datasets before(left) and after(right) immunotherapy.

Factors related to immune regulation play a crucial role in shaping the TME and affecting the effectiveness of cancer immunotherapy (12). A heatmap analysis was performed to explore the relationship between ARPC1A and immune-related molecules across cancers. ARPC1A exhibited significant negative correlations with immune-related genes (MHC, immunosuppressors, immune stimulators, chemokines) in most cancer types (**Figure 5A**). Using the TIMER2.0 resource, the association of ARPC1A expression with the presence of various immune cell types was examined. The analysis indicated a negative correlation between ARPC1A mRNA expression levels and the abundance of B cells and CD8+ T cells in most cancer types, suggesting that ARPC1A may suppress the TME (**Figure 5B**).

We conducted an additional assessment of ARPC1A’s potential impact on cancer immunotherapy by analyzing cancer immune cycle activity scores using the TIP database. An examination of the seven stages of the immune cycle in groups with elevated versus reduced ARPC1A expression revealed that increased ARPC1A levels correlated with lower TIP scores across most cancer types, suggesting possible immunosuppressive effects within the TME (**Figure 5C**). The relationship of ARPC1A mRNA expression with TMB/MSI across various cancer types was examined, taking into account the significance of PD-L1, MSI, and TMB as key biomarkers for immunotherapy. Significant positive correlations were observed between ARPC1A mRNA expression and elevated MSI/TMB scores in BRCA and HNSC (**Figure 5D**). Subsequently, we continued to analyze the potential of ARPC1A expression as a predictor of immune therapy response. First, we examined the changes in ARPC1A expression between the immune response and non-response groups before and after immune therapy across different datasets. In both ESCA and SKCM, ARPC1A expression was significantly elevated in the non-response group post-immune therapy, while no significant differences were observed before treatment (**Figures 5E, F**). Moreover, survival analysis showed that patients with high ARPC1A expression had worse OS and PFS before and after receiving anti-PD1 and/or anti-CTLA4 treatment (**Figures 5G, H**). Overall, the above results emphasize the association between high ARPC1A expression and tumor immune suppression, suggesting its potential as an immunotherapy target and a biomarker for predicting cancer immunotherapy response.

### 2.6 High expression of ARPC1A in NSCLC

**Figure 6:**
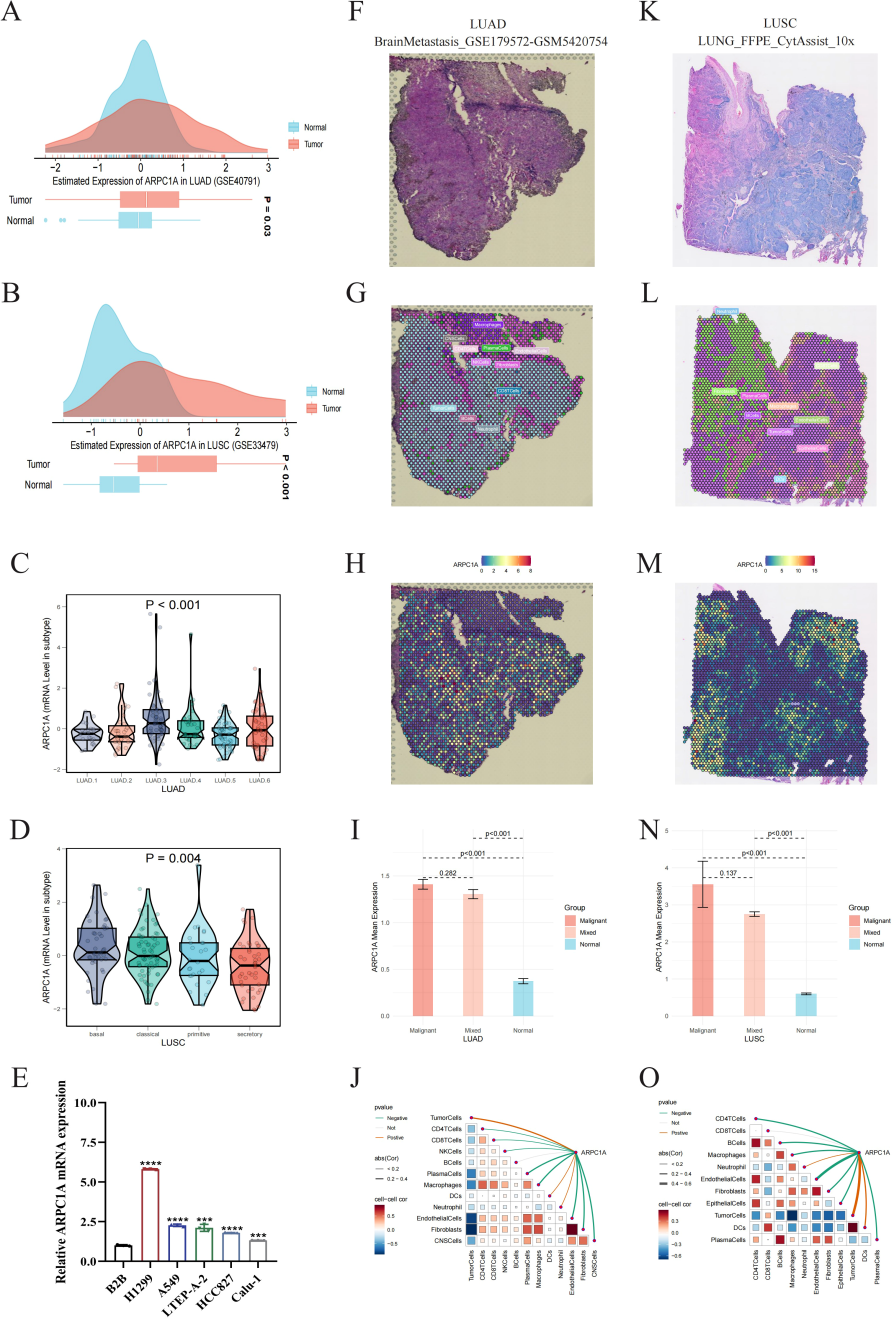
High expression of ARPC1A in NSCLC. **(A, B)** GEO datasets validate high ARPC1A mRNA expression in lung adenocarcinoma (LUAD) and squamous cell carcinoma (LUSC). **(C, D)** ARPC1A expression differences across molecular subtypes of LUAD and LUSC. Kruskal-Wallis Rank Sum Test was used to compare ARPC1A expression among molecular subtypes in TCGA-LUAD and TCGA-LUSC cohorts. **(E)** ARPC1A mRNA expression levels in normal bronchial epithelial cell line BEAS-2B and lung adenocarcinoma cell lines. **(F, K)** High-resolution spatial transcriptomics images of lung adenocarcinoma (BrainMetastasis_GSE179572-GSM5420754) and squamous cell carcinoma (LUNG_FFPE_CytAssist_10x). **(G, L)** Cell component distribution for each spot after spatial transcriptomics deconvolution. Spots are represented by circles; different colors denote different cell types. **(H, M)** Spatial transcriptomics localization of ARPC1A. Spots with redder hues indicate higher ARPC1A expression. **(I, N)** ARPC1A expression differences in malignant (Malignant), mixed malignant (Mixed), and normal (Normal) regions. The x-axis represents different spot types, and the y-axis shows the average ARPC1A expression. Wilcoxon Rank Sum Tests were used to assess statistical differences. **(J, O)** Spearman correlation of ARPC1A expression with microenvironmental components at low resolution. Red curves indicate positive correlations, green curves negative correlations, and gray curves no significant correlation. Thicker lines represent higher correlation coefficients. Red squares denote positive correlations, blue squares negative correlations. Darker colors indicate greater statistical significance, and larger squares denote higher absolute correlation coefficients. ****P < 0.0001, compared with the control group.

Considering the high rates of incidence and mortality associated with NSCLC, along with previous data showing increased ARPC1A expression in LUAD as well as LUSC (**Figures 1A–C**), our next objective was to further assess ARPC1A expression levels in NSCLC. Initially, elevated levels of ARPC1A in LUAD and LUSC were validated using GEO datasets (**Figures 6A, B**). Subsequently, we compared ARPC1A expression across different molecular subtypes of LUAD and LUSC. The ‘LUAD 3’ subtype of LUAD and the ‘basal’ subtype of LUSC exhibited the highest levels of ARPC1A, with differential gene expression observed across various molecular subtypes (**Figures 6C, D**). ARPC1A mRNA expression was consistently high in the NSCLC cell lines H1299, A549, LTEP-A-2, HCC827, and Calu-1, whereas the normal lung epithelial cell line BEAS-2B showed lower levels (**Figure 6E**). Finally, we validated ARPC1A expression in LUAD and LUSC using spatial transcriptomics. Analysis of tissue sections from LUAD and LUSC (**Figures 6F, K**) and the spatial distribution of major cell types (**Figures 6G, L**) revealed that ARPC1A expression closely correlates with the localization of tumor cells (**Figures 6H, M**). Quantification showed higher ARPC1A expression in malignant regions, suggesting predominant expression in tumor cells (**Figures 6I, N**). In line with earlier localization findings, a significant positive correlation was observed between ARPC1A expression levels and the tumor cell content within the spots. In conclusion, these findings suggest that ARPC1A exhibits high expression levels in NSCLC.

### 2.7 Knockdown of ARPC1A suppresses malignant phenotypes in NSCLC cells

**Figure 7:**
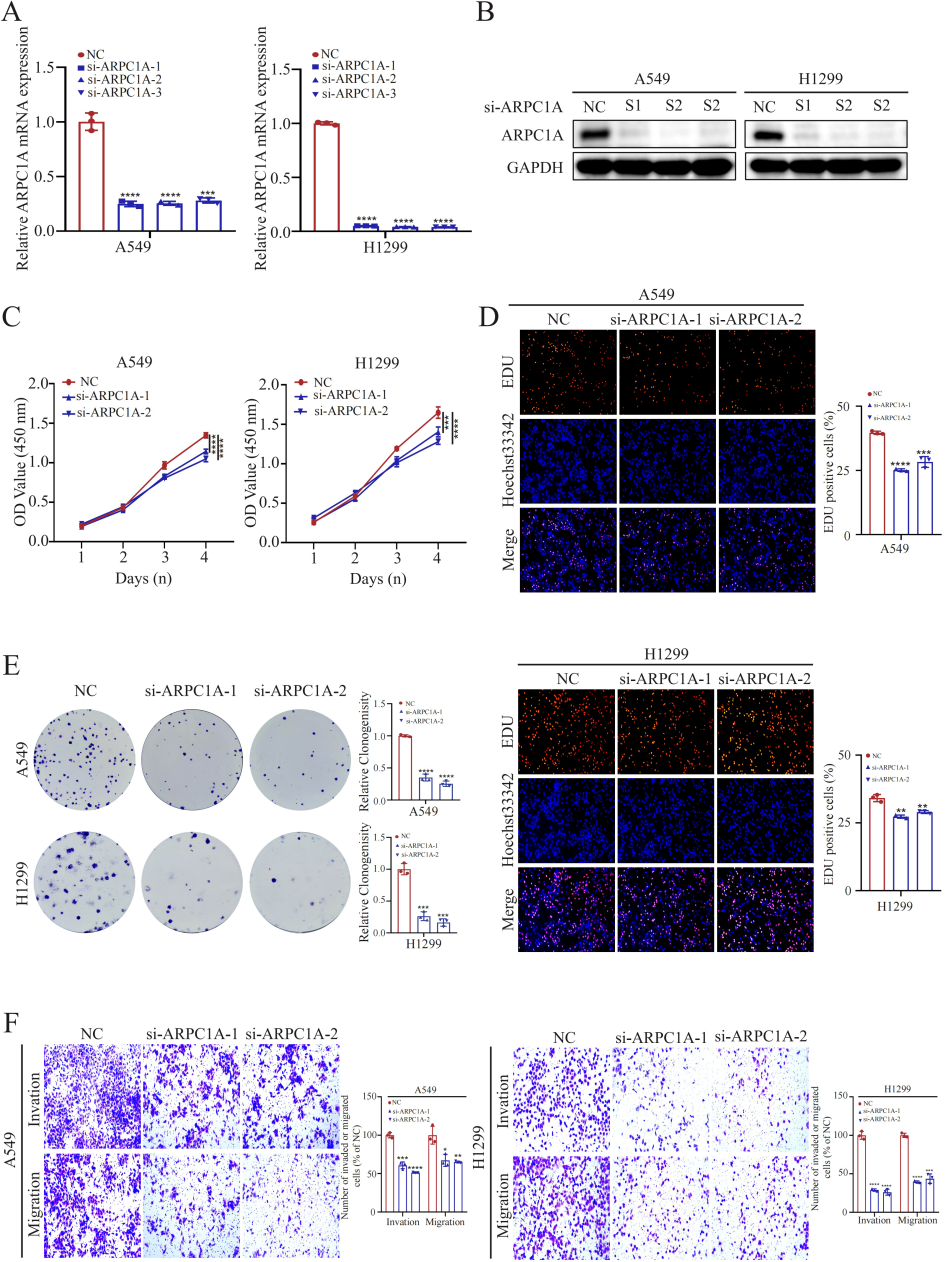
Knockdown of ARPC1A Suppresses Malignant Phenotypes in NSCLC Cells. **(A, B)** Validation of si-ARPC1A knockdown efficiency using qPCR and Western blotting. **(C–E)** Assessment of cell proliferation changes using CCK-8, EdU, and colony formation assays. **(F)** Transwell assay to evaluate cell invasion and migration capabilities. *P < 0.05, **P < 0.01, ***P<0.001, ****P<0.0001, compared with the control group.

Initially, we screened and validated siRNA sequences targeting ARPC1A using qPCR and Western blot analyses (**Figures 7A, B**). The influence of reducing ARPC1A expression on the malignancy of NSCLC cells was subsequently examined. The growth of H1299 and A549 cells was significantly reduced by ARPC1A silencing, as revealed by CCK-8, Edu incorporation, and colony formation assays (**Figures 7C–E**). Moreover, the invasive and migratory abilities were notably diminished by ARPC1A silencing, as confirmed by Transwell assays (**Figure 7F**). Overall, these results indicate that ARPC1A contributes to tumorigenesis in NSCLC.

### 2.8 ARPC1A may promote NSCLC progression by regulating c-Myc expression

**Figure 8:**
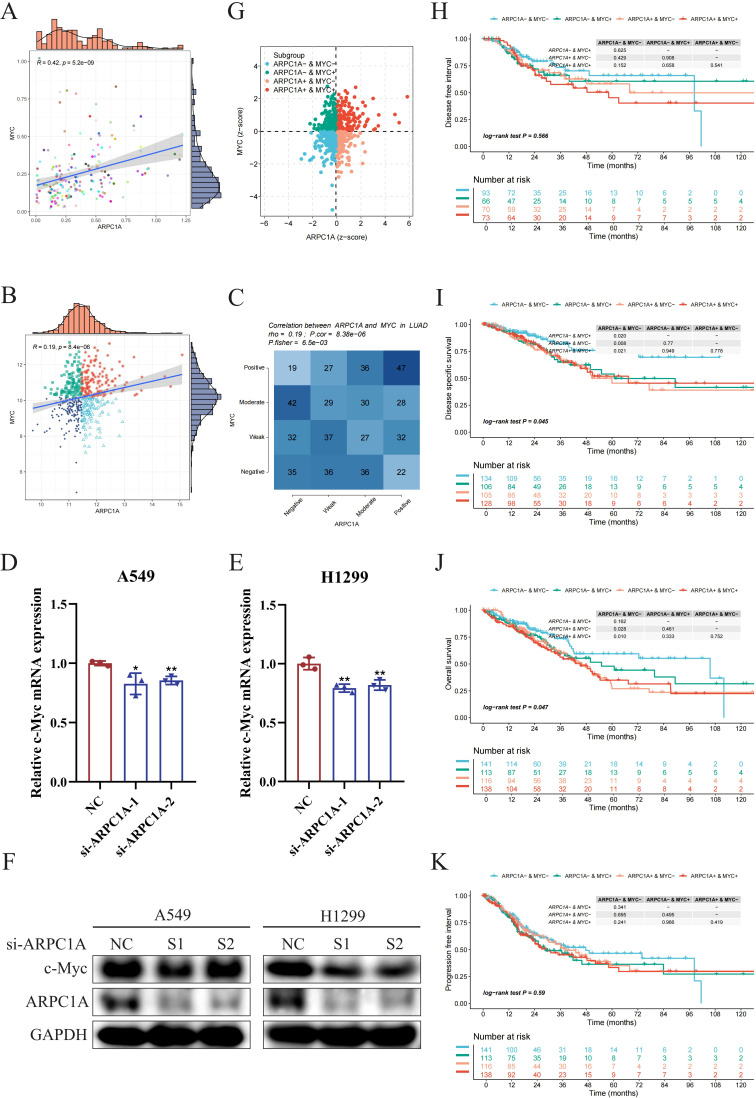
ARPC1A may promote NSCLC progression by regulating c-Myc expression. **(A)** Correlation analysis of expression levels based on single-cell datasets. The x-axis and y-axis represent the average expression levels of ARPC1A and c-Myc in each single-cell dataset, respectively. Each scatter point represents an individual dataset, with colors indicating different datasets. Spearman’s correlation coefficient (R) and p-value are shown, with p < 0.05 considered significant. **(B)** Correlation analysis based on transcriptomic data. Samples are categorized into ARPC1A/MYC high expression, ARPC1A high expression/MYC low expression, ARPC1A low expression/MYC high expression, and ARPC1A/MYC low expression groups based on median expression levels of ARPC1A and MYC. Different colors and shapes of scatter points represent these groups. **(C)** Correlation analysis and Fisher’s exact test. Genes are classified into four categories (Positive, Moderate, Weak, Negative) based on expression levels. A contingency table heatmap visualizes this, with color intensity indicating sample quantity. Darker diagonal squares denote higher absolute correlation coefficients between genes. **(D–F)** qPCR and Western blotting to detect changes in c-Myc expression following ARPC1A knockdown. **(G)** Z-score scatter plot for ARPC1A and MYC samples. Each point represents a sample, with colors denoting different subgroups. The x- and y-axes represent the Z-scores of ARPC1A and MYC, respectively; Z-score ≤ 0 indicates low expression, and Z-score > 0 indicates high expression. **(H–K)** Kaplan-Meier survival analysis for ARPC1A/c-Myc. The x-axis represents survival time (t), and the y-axis denotes the probability of survival beyond time t. Log-rank test assesses differences between survival curves, with p < 0.05 considered significant. A table with a gray background shows Log-rank test results for pairwise comparisons, with the overall Log-rank test result in the lower-left corner. *P < 0.05, **P < 0.01, compared with the control group.

Given that previous GSEA results suggested ARPC1A might activate the MYC signaling pathway, we aimed to verify whether ARPC1A regulates c-Myc expression. Initially, we assessed the correlation between ARPC1A and c-Myc expression using single-cell datasets and TCGA-based transcriptomic data. Single-cell data revealed a strong positive correlation between ARPC1A and MYC expression, suggesting a potential co-expression relationship (**Figure 8A**). The analysis of transcriptomic data provided support for this observation (**Figures 8B, C**). Notably, qPCR and Western blot analyses confirmed that ARPC1A knockdown downregulated c-Myc expression at both transcript and protein levels (**Figures 8D–F**). Finally, survival data for ARPC1A/c-Myc were compared to further validate their association within the same signaling axis. Results showed that the ARPC1A+/MYC+ group had worse OS and DSS compared to the SYAP1-/MKI67- group, while no significant differences were observed for DF1 and PFI (**Figures 8G–K**). Collectively, these results suggest that ARPC1A’s oncogenic role in NSCLC may be mediated through c-Myc.

### 2.9 ARPC1A is a potential chemotherapy resistance gene

**Figure 9:**
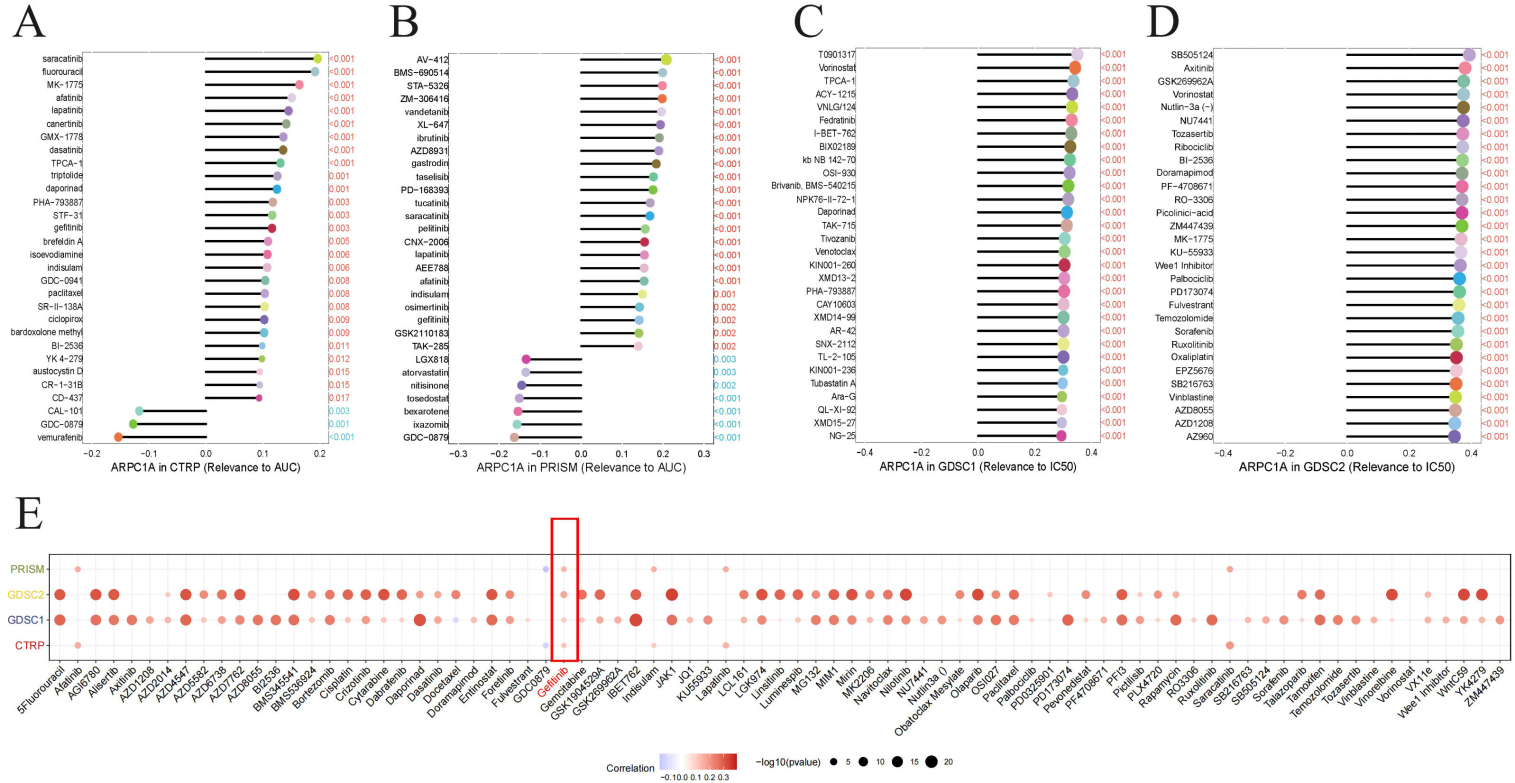
ARPC1A is a potential chemotherapy resistance gene. **(A–D)** Association between ARPC1A mRNA expression and chemotherapy drug sensitivity. The x-axis shows the Spearman correlation coefficient between drug IC50 or AUC values and gene expression, while the y-axis lists the top 30 drugs with the most significant p-values. Each drug is depicted as a lollipop plot: red indicates a positive correlation with ARPC1A, and blue indicates a negative correlation. Longer lollipops represent higher correlation coefficients. **(E)** Heatmap summarizing drug resistance related to ARPC1A from panels **(A–D)**.

Recent research indicates that chemotherapy-resistant cancer stem cells and tumor cells with primary or acquired resistance to targeted therapies exhibit a high dependence on oxidative phosphorylation (13–15). Notably, MYC can enhance oxidative phosphorylation to promote chemotherapy resistance in breast cancer (16). Given previous GSEA results suggesting that ARPC1A may activate the oxidative phosphorylation signaling pathway and *in vitro* evidence showing that ARPC1A regulates c-Myc expression, we hypothesize that ARPC1A may be a potential chemotherapy resistance gene. The relationship of drug sensitivity with ARPC1A mRNA levels was analyzed using three separate databases [CTRP (17), GDSC (18), and Cellminer (19)] to explore the link of ARPC1A and chemotherapy resistance. The results suggest that ARPC1A is a potential chemotherapy resistance gene (**Figures 9A–D**). Importantly, data from all three databases indicate that ARPC1A is associated with resistance to gefitinib (**Figures 9E**), implying that targeting ARPC1A could improve treatment outcomes for gefitinib-resistant cancer patients.

## 3 Discussion

Extensive research highlights the critical role of the Arp2/3 complex in tumor cell invasion and migration through its regulation of cellular pseudopodia formation and cytoskeletal dynamics. Targeting the Arp2/3 complex has become a major focus in anti-tumor invasion therapies. Recent studies show that inhibiting the Arp2/3 complex subunit ARPC2 can suppress cancer cell migration while sparing normal cells. Notably, two ARPC2-binding inhibitors have been FDA-approved for clinical use. Although targeting ARPC2 modulates Arp2/3 complex activity, its composition varies across different cancers, with differential expression levels of each subunit observed in various tumors. Research by Cao et al. demonstrates that the Arp2/3 complex inhibitors CK-666 and CK-869 inhibit actin branching in a manner dependent on Arp2/3 iso-complex composition (20). This underscores the need to elucidate subunit expression levels across different cancers for the development of targeted small-molecule inhibitors as therapeutic strategies.

Gene expression analysis (**Figure 1**) demonstrates that subunits of the Arp2/3 complex are markedly upregulated in various tumor types relative to normal tissues and are associated with unfavorable outcomes in certain cancers. Evidence supports the association between certain subunits and tumor progression. For instance, it was demonstrated by Huang et al. that elevated ARPC1B expression in ovarian cancer facilitates tumor development through the Wnt/β-catenin signaling pathway (21). Mei et al. found that EVs with high ARPC2 expression enhance proliferation and migration of hepatocellular carcinoma cells (22). Additionally, our research suggests that dysregulation of Arp2/3 complex subunits may be associated with copy number variations. However, in certain tumors such as THYM and UVM, the correlation is weak. Therefore, further bioinformatic analysis across pan-cancer datasets to explore the potential association between DNA methylation, protein phosphorylation, and other factors (23, 24) with the imbalance in the expression of Arp2/3 complex subunits is warranted.

Given the oncogenic potential of Arp2/3 complex subunits, CMap analysis was used to identify small molecules that interact with these subunits across different cancer types. Among these, 4,5-Dianilinophthalimide emerges as a potential agent interacting with most Arp2/3 complex subunits. Previous studies indicate that 4,5-Dianilinophthalimide can inhibit the tyrosine kinase activity of EGF-R (25). Based on subunit expression levels, in CHOL, where ARPC1B is highly expressed, the top three small molecules are MK-886, 4,5-Dianilinophthalimide, and clofibrate. MK-886, an ALOX5 inhibitor with anti-inflammatory and antioxidant properties, significantly inhibits HepG2 cell proliferation and induces cell death in a dose-dependent manner (26).

A crucial component of the Arp2/3 complex, ARPC1A, plays a role in controlling actin polymerization. Given its high expression in most tumor types and significant diagnostic value, we selected ARPC1A for further pan-cancer functional analysis and experimental validation. GSEA enrichment analysis revealed significant activation of pathways related to oxidative phosphorylation and cell proliferation, including the MYC signaling pathway. Significantly, we verified elevated ARPC1A expression in cell lines of NSCLC. Marked suppression of growth, invasion, and migration in lung adenocarcinoma cells is observed when ARPC1A expression is reduced, likely mediated by c-Myc. Furthermore, GSEA enrichment results suggest that ARPC1A may inhibit immune-related pathways, including interferon (IFN)-γ. ARPC1A’s potential as a biomarker for predicting immune responses across various cancers is suggested by further analysis of its association with immune cell infiltration and immune-related regulatory factors. Notably, recent studies have highlighted the significant value of machine learning (ML) in building cancer diagnostic and prognostic models, as well as in predicting immune therapy outcomes (27–31). Future research should further explore the potential diagnostic and therapeutic efficacy of Arp2/3 complex subunits across pan-cancer using ML, aiming to identify optimal biomarkers and immune therapy targets.

In summary, our study reveals that Arp2/3 complex subunits are overexpressed in most tumor types relative to normal tissues and are linked to poor prognosis in certain cancers. Copy number variations may contribute to this expression imbalance. Specifically, ARPC1A appears to enhance cell proliferation and oxidative phosphorylation across various cancers through the regulation of c-Myc expression. Crucially, our results emphasize the potential of ARPC1A as a new biomarker for diagnosing and predicting tumors, along with its viability as a therapeutic target. However, additional studies are required to investigate and elucidate the specific molecular mechanisms through which ARPC1A regulates cancer progression and tumor immunity. Future studies will be crucial for further validating these findings and advancing their clinical applications. First, large-scale clinical cohorts should be utilized to assess the diagnostic value of ARPC1A across different cancer types and its correlation with clinical features, such as tumor staging and prognosis. Second, the role of ARPC1A in the tumor immune microenvironment suggests its potential as a target for immune therapy. Clinical trials targeting ARPC1A as a potential therapeutic combination with immune therapies could open new avenues for cancer immunotherapy. Finally, small molecule inhibitors or antibodies targeting ARPC1A could become powerful tools for combating tumor metastasis. Future research should focus on developing targeted therapeutic strategies against ARPC1A, while exploring their safety and efficacy in clinical applications.

## 4 Materials and methods

### 4.1 Assessment of Arp2/3 complex subunit levels across cancers

Wilcoxon rank-sum tests were conducted on TCGA data to compare mRNA levels of Arp2/3 complex subunits between tumor and non-cancerousl tissues, as well as within paired samples across different cancer types, with significance determined at p < 0.05. Additionally, we expanded the normal tissue sample size using information provided by GTEx to enhance confidence in our results.

### 4.2 Spatial transcriptomics analysis of ARPC1A in NSCLC

A deconvolution analysis method based on spatial and single-cell transcriptomics data was applied to assess the cellular composition at each spot on the 10x Visium slides (32, 33). The SpatialFeaturePlot function from Seurat was used to visualize the enrichment scores for each cell type. Spots are represented by circles, with colors indicating different cell types; deeper colors reflect higher enrichment scores and a higher content of that cell type. A malignant score of 1 classifies a spot as Malignant, 0 as Normal, and intermediate scores as Mixed.

### 4.3 Clinical utility of Arp2/3 complex subunits in pan-cancer

The significance of Arp2/3 complex subunits in early pan-cancer diagnosis was evaluated using the receiver operating characteristic (ROC) curve metrics computed by the ‘pROC’ R package. A value of the ROC curve closer to 1 signifies superior diagnostic performance. Univariate Cox proportional hazards analysis for prognostic assessment was carried out using the ‘survival’ package. Results of the univariate Cox analysis were visualized using heatmaps.

### 4.4 Alterations in somatic genomic copy number and mutations of Arp2/3 complex subunits

Data on somatic variants and DNA copy number alterations (CNA) for a pan-cancer analysis were retrieved from the cBioPortal website (34). The Spearman correlation between the expression levels of Arp2/3 complex subunits and DNA copy number alterations was calculated to assess the association of SCNA with Arp2/3 complex subunit expression. Results were presented as a heatmap.

### 4.5 Assessment of pathway activity for Arp2/3 complex subunits

Pathway activity was assessed using the GSVA package with four methods (z-score, gsva, ssGSEA, plage) (35, 36). To standardize the data, scores from all methods except z-score were transformed to unitless Z-scores by (x-μ)/σ for each tumor. Variations in statistical measures between tumor and healthy tissues were evaluated using the Wilcoxon-Mann-Whitney test. Boxplots generated using the ggplot2 library were used to visualize the results.

### 4.6 Investigating the possible biological roles of ARPC1A in pan-cancer

The classification of patients from the TCGA dataset into high and low ARPC1A expression groups relied on their ARPC1A expression levels. Ans the levels of stimulation or suppression of 50 fundamental gene sets and 83 metabolic pathway gene sets across differing expression levels in various tumors were assessed using GSEA (37). In addition, the connection linking ARPC1A mRNA levels and protein expression measured by RPPA in the TCPA database (38), was assessed using Rank-based association analysis. Results across all tumors were visualized using heatmaps.

### 4.7 Pan-cancer analyses of the immunological roles of ARPC1A

To assess MeTIL characteristics, Methylation levels of MeTIL markers were transformed into MeTIL scores using principal component analysis (PCA). Z-scores were calculated for the data using the formula (x-μ)/σ, and elevated and reduced levels were determined according to the median ARPC1A level. Wilcoxon Rank Sum Tests were used to assess statistical differences in MeTIL scores between conditions. The relationship of ARPC1A with various immunomodulators and chemokines, including immune stimulatory genes, immunosuppressive genes, chemokines, and human leukocyte antigens, was investigated using the TISIDB platform. Results were visualized using a heatmap. Using TIMER2.0 (39) and 7 sophisticated methods (XCELL,CiberSort_ABS, CiberSort, EPIC, QUANTISEQ, MCPCOUNTER, TIMER), we assessed the effect of ARPC1A expression on immune cell infiltration across various TCGA cancer types. In addition, we used the GSVA algorithm to assess differences in TIPs scores between elevated and reduced ARPC1A expression groups.

### 4.8 Determining chemical substances that bind to the subunits of the Arp2/3 complex

Spearman correlation analysis was conducted to explore how AUC values from the CTRP and PRISM databases, along with IC50 values for antagonists from the GDSC1 and GDSC2 databases, relate to ARPC1A expression. A negative correlation indicates increased drug sensitivity with higher ARPC1A expression, while a positive correlation indicates reduced sensitivity. cMAP analysis was used to identify potential compounds that counteract the pro-cancer effects mediated by Arp2/3 complex subunits. The XSum (eXtreme Sum) method for precise feature alignment was employed to assess gene-related features against cMAP gene signatures, yielding correlation values across 1,288 substances. Compounds with lower scores may inhibit the pro-cancer effects induced by Arp2/3 complex subunits.

### 4.9 Culturing cells and performing transfections

DMEM with 10% FBS (Gibco, USA) was used to culture BEAS-2B normal bronchial epithelial cells. RPMI 1640 medium with 10% FBS was used to culture the human NSCLC cell lines LTEP-A-2, H1299, HCC827 and A549.​​ Lipofectamine 3000 (Thermo, USA) was used for *in vitro* transfection in accordance with the instructions provided by the manufacturer. siRNA sequences targeting ARPC1A were as follows: si-ARPC1A-1 (5’-GGUGGAGCAAGCAUGUUAA-3’), si-ARPC1A-2 (5’-GGUGGAGCAAGCAUGUUUU-3’), and si-ARPC1A-3 (5’-CCAAGUUGAUUCUUGGAAAG-3’).

### 4.10 Total RNA extraction and quantitative PCR

An RNA extraction kit (Invitrogen, CA, USA) was used to extract total RNA from cell lines. Quantitative PCR (qPCR) was conducted using an ABI Q5 PCR system (Applied Biosystems, CA, USA). The 2^(-ΔΔCt) method was used to assess the data, with β-actin used as the reference gene. The primer sequences: β-actin (Forward, 5’-GACGGCTACCCGATCTCGGCAT-3’; Reverse, 5’-ACGGCTTTCCAGCGCATCCGCA-3’.); ARPC1A(Forward, 5’-ATTGCCCTCAGTCCCAATAATCA-3’; Reverse, 5’-CAAGTGACAATGCGGTCGC-3’).

### 4.11 CCK-8 assay

Cell viability was assessed according to the reagent kit instructions(Beyotime, China). Briefly, 2800 cells were seeded per well in a 96-well plate, and absorbance at 450 nm was measured daily at fixed time points after cell attachment.

### 4.12 EDU assay

EDU assays were conducted according to the manufacturer’s instructions (Beyotime, China). After staining, images were captured under a microscope and cell counts were analyzed using Image J.

### 4.13 Colony formation assay

200 cells transfected for 24 hours were added to each well of a six-well plate. Cultures were terminated when visible cell clusters formed under the microscope. Cells were fixed with 4% paraformaldehyde and stained with crystal violet for 20 minutes. Images were captured and cell clusters were quantified using Image J.

### 4.14 Western blot

Cells were lysed with RIPA buffer, and the protein concentration in the supernatant was measured. Twenty micrograms of protein per well were resolved by SDS-PAGE and blotted onto PVDF membranes. Blocking was done with a commercial solution for 10 minutes. The primary antibody was incubated at 4°C for 15 hours, followed by secondary antibody incubation at room temperature for 1 hour, and then developed.

### 4.15 Transwell assay

Cell migration and invasion assays were performed using Transwell chambers (Corning, MD, USA). For the migration assay, 20,000 cells in serum-free 1640 medium were added to the lower chamber, and 200 µL of 20% serum-containing 1640 medium was added to the upper chamber. Cells were fixed and stained with crystal violet after 24 hours. In the invasion assay, Matrigel was added to the upper chamber, and the incubation was extended to 36 hours. All other procedures were identical to those used in the migration assay.

### 4.16 Statistical analysis

Web-based tools and R version 4.3.0 were used to process all data. Pearson’s r was used for data with a normal distribution, whereas Spearman’s rank correlation was employed for data that was not normally distributed. Comparisons between two variables were assessed using the Wilcoxon signed-rank test and the Wilcoxon rank-sum test, respectively. The Kruskal-Wallis test was used to analyze variations among multiple variables. The ‘proc’ R package was used to conduct ROC analysis for assessing the diagnostic capability of Arp2/3 complex subunits. A p-value of less than 0.05 indicated statistical significance, while high significance was represented by a p-value of less than 0.0001 (*p < 0.05, **p < 0.01, ***p < 0.001, ****p < 0.0001).

## 5 Conclusion

In summary, our study supports targeting Arp2/3 complex subunits as a novel anticancer strategy, highlights the prognostic and diagnostic value of ARPC1A across cancers, and emphasizes the connections between ARPC1A, oxidative phosphorylation, and tumor immunity.

## Data Availability

The datasets presented in this study can be found in online repositories. The names of the repository/repositories and accession number(s) can be found in the article/**Supplementary Material**.
