# Supplementary material for: Pan-cancer analysis of Arp2/3 complex subunits: focusing on ARPC1A’s role and validating the ARPC1A/c-Myc axis in non-small cell lung cancer
Source: Front Immunol. 2025 Jan 10;15:1491910. doi: 10.3389/fimmu.2024.1491910 (PMC11759278; doi:10.3389/fimmu.2024.1491910)
Supplement: Supplementary file 1 [file DataSheet1.docx]

Supplementary Material

# Supplementary Figures

##
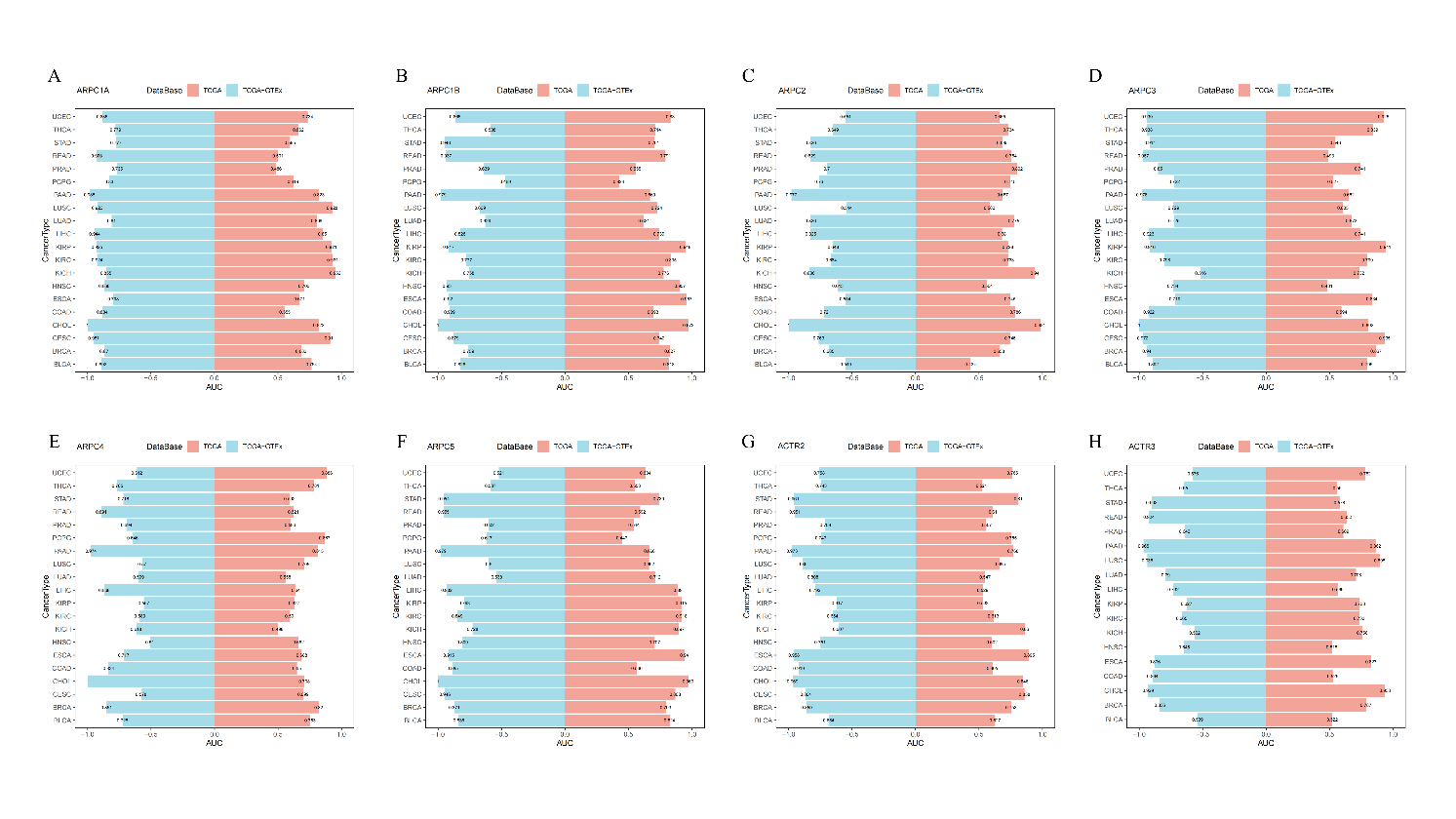
Supplementary Figure 1

**Supplementary Figure 1.** **Diagnostic values of Arp2/3 complex subunits.** (A-H) Diagnostic value of Arp2/3 complex subunits in differentiating tumor from normal tissue: a pan-cancer perspective using TCGA and TCGA-GTEx data.

## Supplementary Figure 2


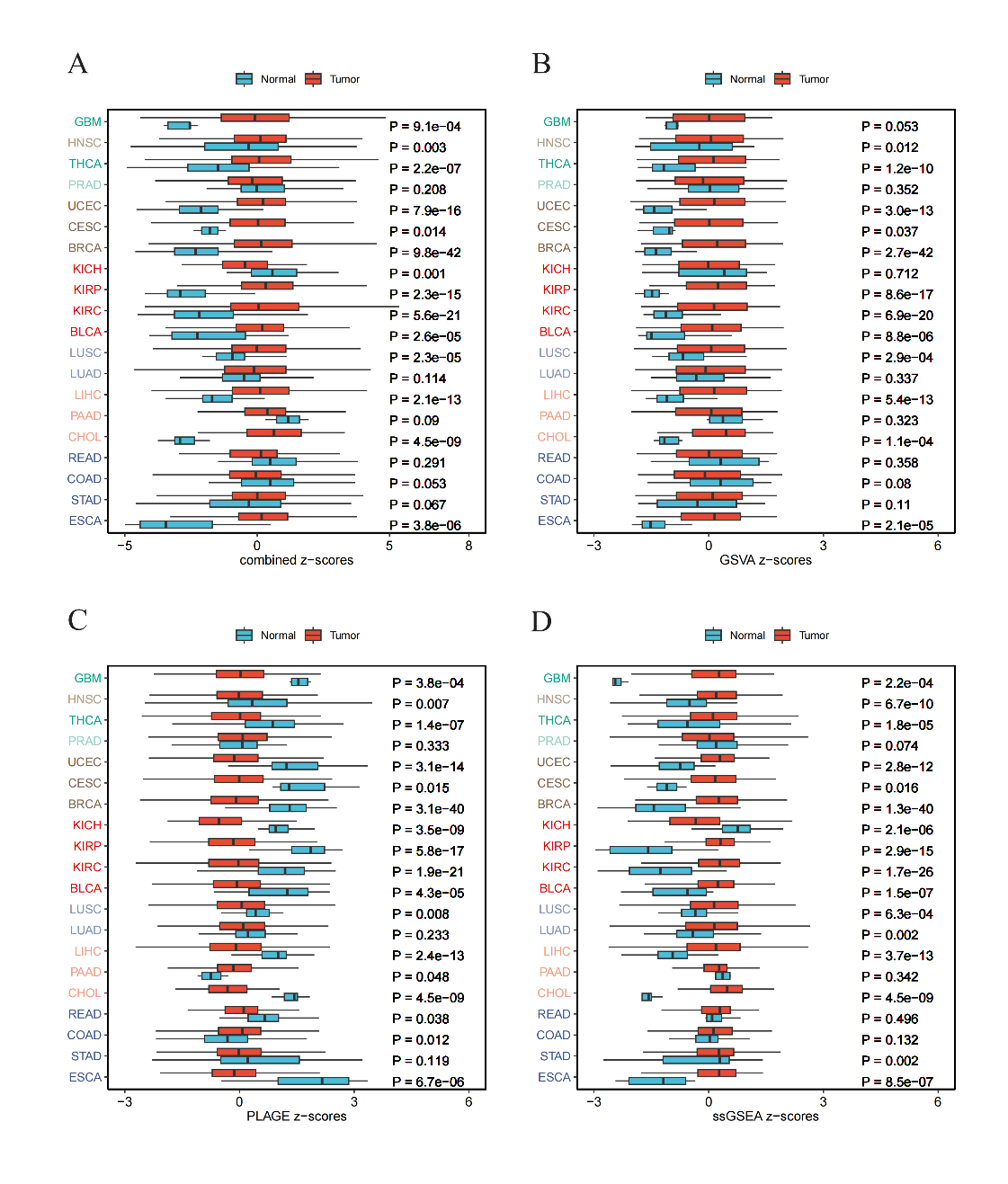


**Supplementary Figure 2.** **Pathway activity for Arp2/3 complex subunits in pan-cancer.** (A-D) Statistical differences between tumor and normal tissues were assessed using Wilcoxon Rank Sum Tests. The ends of the boxes represent the interquartile range, with the line inside the box indicating the median.

## Supplementary Figure 3


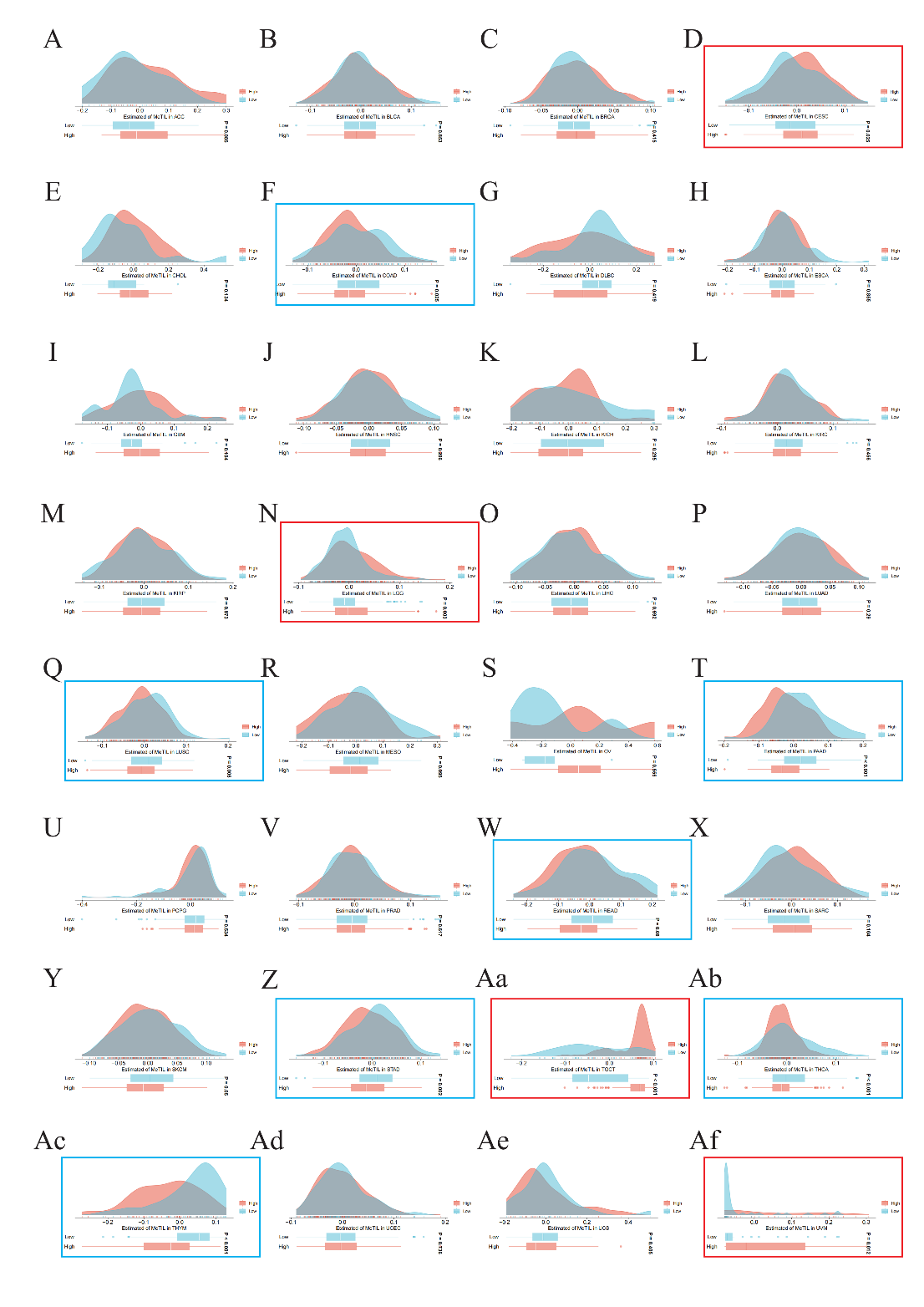


**Supplementary Figure 3.** **Differences in MeTIL scores between high and low ARPC1A expression groups.** (A-Af) The top of the figure shows the distribution of MeTIL levels for individual samples in the high and low expression groups. The ends of the boxes represent the interquartile range, with the line inside the box indicating the median. Wilcoxon Rank Sum Tests were used to assess statistical differences between the two groups.
