# Supplementary material for: Pan-cancer analysis of Arp2/3 complex subunits: focusing on ARPC1A’s role and validating the ARPC1A/c-Myc axis in non-small cell lung cancer
Source: Front Immunol. 2025 Jan 10;15:1491910. doi: 10.3389/fimmu.2024.1491910 (PMC11759278; doi:10.3389/fimmu.2024.1491910)
Supplement: Supplementary file 2 [file DataSheet2.pdf]

Figure 7 B A549

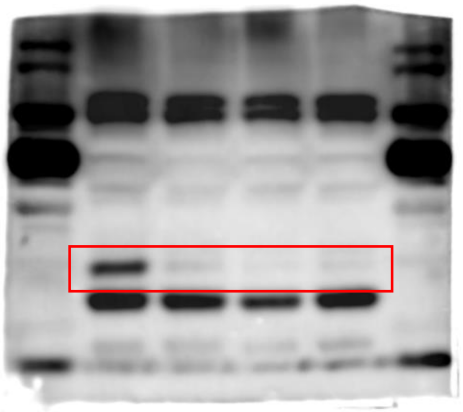

ARPC1A

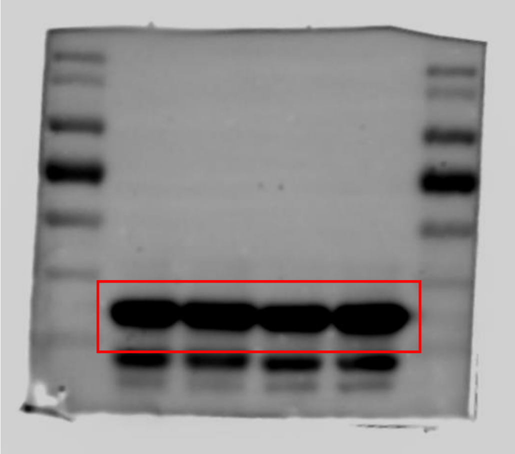

GAPDH

Figure 7 B H1299

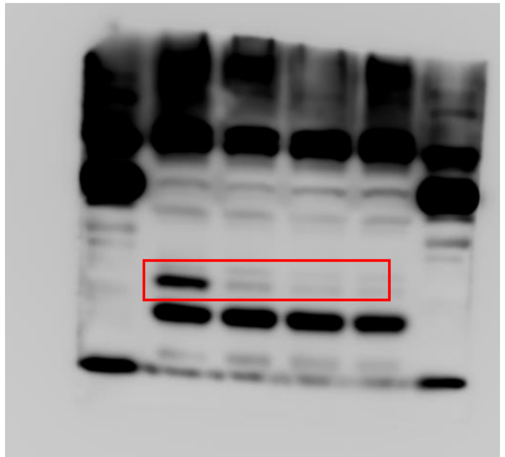

ARPC1A

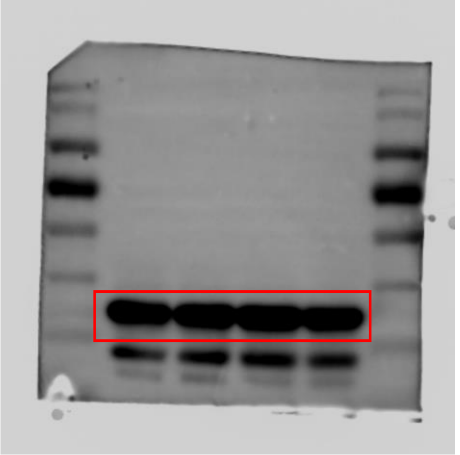

GAPDH

Figure 8 F A549

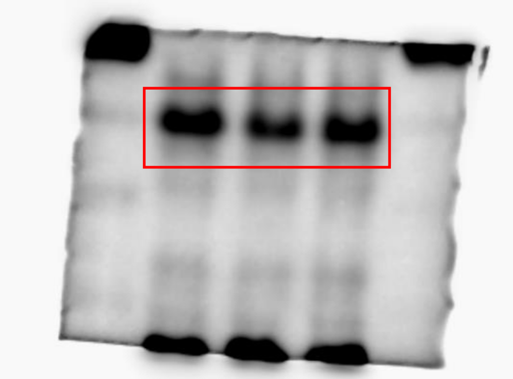

c-Myc

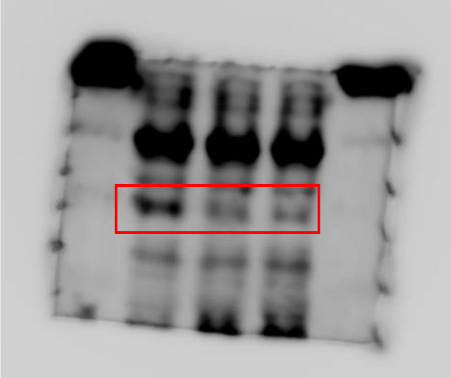

ARPC1A

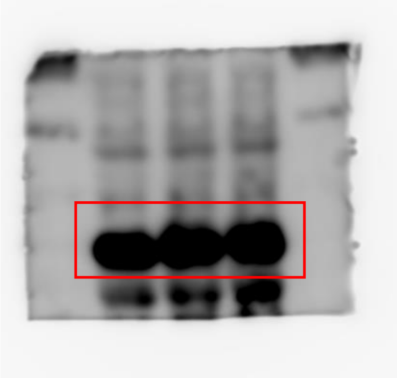

GAPDH

Figure 8 F H1299

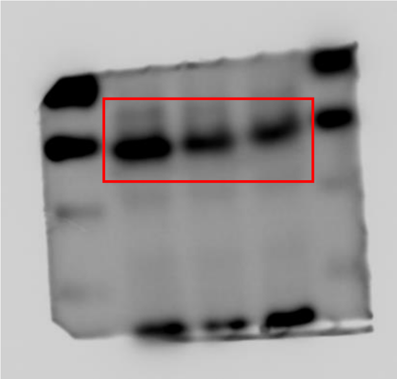

c-Myc

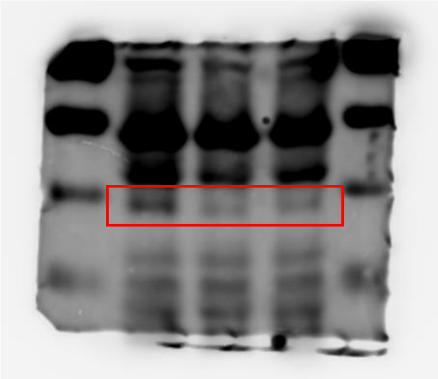

ARPC1A

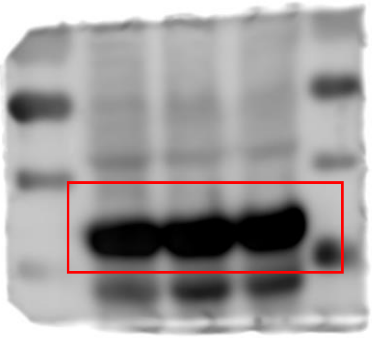

GAPDH
